# Complete genome sequence of *Serratia marcescens* MPHSM1, isolated from a patient with necrotizing fasciitis

**DOI:** 10.1128/mra.00634-25

**Published:** 2025-10-10

**Authors:** Mako Mamiya, Akira Sata, Toshiki Inoue, Debora Satie Nagano, Yumi Imai, Atushi Yamanaka, Yoshitoshi Ogura

**Affiliations:** 1 Department of General Medicine, Miyazaki Prefectural Miyazaki Hospital 13610 , Miyazaki, Japan; 2 Department of Clinical Laboratory, Miyazaki Prefectural Miyazaki Hospital 13610 , Miyazaki, Japan; 3 Division of Microbiology, Department of Infectious Medicine, Kurume University School of Medicine 26333 , Fukuoka, Japan; University of Maryland School of Medicine, Baltimore, Maryland, USA

**Keywords:** *Serratia marcescens*, necrotizing fasciitis, complete genome sequence

## Abstract

*Serratia marcescens* is an opportunistic pathogen that causes a variety of hospital-acquired and community-acquired infections. Here, we report the complete genome sequence of *S. marcescens* strain MPHSM1 responsible for necrotizing fasciitis. The genome is 5,022,671 bp long, with a G+C content of 59.76%.

## ANNOUNCEMENT

*Serratia marcescens* is a Gram-negative, rod-shaped opportunistic pathogen within the *Yersiniaceae* family, typically isolated from both human clinical samples and environmental sources. In humans, it is associated with a range of infections, including both community-acquired and nosocomial infections, such as bloodstream infections, skin infections, respiratory tract infections, and urinary tract infections (1). Furthermore, *S. marcescens* occasionally induces necrotizing fasciitis, a rapidly progressive and severe soft tissue infection, particularly in individuals with underlying risk factors such as diabetes mellitus and hepatic dysfunction (2). We isolated the *S. marcescens* strain MPHSM1 from the blood of a 58-year-old female patient with chronic heart failure who developed necrotizing fasciitis and ultimately succumbed to the infection.

Blood cultures were performed using the BACTEC FX system (Becton Dickinson Japan Ltd.) with BD BACTEC 22F anaerobic and 23F aerobic resin bottles, incubated at 35°C. After 9 h, both aerobic and anaerobic bottles became positive. Positive specimens were subcultured on sheep blood agar and BTB lactose agar and incubated aerobically at 35°C for 24 to 48 h. Colonies with hemolysis and red pigmentation were observed the following day.

Genomic DNA was extracted from MPHSM1 using the Genomic-tip 100/G and Genomic DNA buffer set (Qiagen) according to the manufacturer’s instructions, after overnight cultivation in LB medium at 37°C. The library for short-read sequencing was prepared using the xGen DNA Library Prep EZ Kit (Integrated DNA Technologies) and NEBNext Multiplex Oligos for Illumina (96 Unique Dual Index Primer Pairs) (New England BioLabs). The library was subjected to sequencing on an Illumina MiSeq platform to generate 301-bp paired-end reads. For long-read sequencing, the library was prepared using Rapid Barcoding Sequencing kit (Oxford Nanopore Technologies). The library was loaded onto the R9.4.1 flow cell on a MinION Mk1C platform. Base calling was performed using Guppy GPU v. 3.4.5 (Oxford Nanopore Technologies). The Illumina run yielded 3,526,866 reads totaling 1,060,224,068 bp, while the nanopore run produced 103,229 reads totaling 738,869,167 bp. Reads were quality-trimmed using Platanus_trim v. 1.1.0 (3) and NanoFilt v. 2.8.0 (4), respectively. After trimming, 1,710,860 short-read pairs (total: 833,075,082 bp) and 26,573 long reads (total: 395,018,345 bp, N50: 14,857 bp) were retained. The assembly and polishing of the combined long reads and short reads data were conducted using MicroPIPE v. 0.9 (5). Default parameters were used for all software unless otherwise specified.

The complete genome of *S. marcescens* MPHSM1 comprises a single circular chromosome, devoid of plasmids, with a length of 5,022,671 bp and a G+C content of 59.76%. Genome annotation was performed using PGAP (6), which identified 4,680 coding sequences, in addition to 22 rRNA genes, 91 tRNA genes, and 1 tmRNA gene. Analysis using ResFinder-2.1 (7) revealed the absence of acquired antimicrobial resistance genes within the MPHSM1 genome. A BLAST search against the Virulence Factor Database VFDB (8) identified a hemolysin family protein, in addition to the well-characterized hemolysin ShlA (9). The complete genome sequence of MPHSM1 will be invaluable for comparative genomic studies and for elucidating the genomic characteristics of *S. marcescens* strains associated with necrotizing fasciitis.

## Data Availability

The genome sequence and annotation data for *S. marcescens* MPHSM1 were deposited under GenBank accession CP182810. Raw sequencing data were deposited in the NCBI Sequence Read Archive (SRA) database under BioProject no. PRJNA1225926 and BioSample no. SAMN46910869. The SRA accession numbers are SRR32409287 (Illumina) and SRR32409286 (ONT).
